# Allopregnanolone Alters the Gene Expression Profile of Human Glioblastoma Cells

**DOI:** 10.3390/ijms19030864

**Published:** 2018-03-15

**Authors:** Carmen J. Zamora-Sánchez, Aylin del Moral-Morales, Ana M. Hernández-Vega, Valeria Hansberg-Pastor, Ivan Salido-Guadarrama, Mauricio Rodríguez-Dorantes, Ignacio Camacho-Arroyo

**Affiliations:** 1 Unidad de Investigación en Reproducción Humana, Instituto Nacional de Perinatología-Facultad de Química, Universidad Nacional Autónoma de México (UNAM), 04510 Mexico City, Mexico; carmenjzamora@gmail.com (C.J.Z.-S.); aylindmm@hotmail.com (A.d.M.-M.); anahdzvg@gmail.com (A.M.H.-V.); 2 Departamento de Biología, Facultad de Química, Universidad Nacional Autónoma de México (UNAM), 04510 Mexico City, Mexico; valeriahp@gmail.com; 3 Instituto Nacional de Medicina Genómica, 14610 Mexico City, Mexico; silvervann@gmail.com (I.S.-G.); mrodriguez@inmegen.gob.mx (M.R.-D.)

**Keywords:** allopregnanolone, progesterone metabolites, finasteride, astrocytomas, glioblastomas

## Abstract

Glioblastomas (GBM) are the most frequent and aggressive brain tumors. In these malignancies, progesterone (P4) promotes proliferation, migration, and invasion. The P4 metabolite allopregnanolone (3α-THP) similarly promotes cell proliferation in the U87 human GBM cell line. Here, we evaluated global changes in gene expression of U87 cells treated with 3α-THP, P4, and the 5α-reductase inhibitor, finasteride (F). 3α-THP modified the expression of 137 genes, while F changed 90. Besides, both steroids regulated the expression of 69 genes. After performing an over-representation analysis of gene ontology terms, we selected 10 genes whose products are cytoskeleton components, transcription factors, and proteins involved in the maintenance of DNA stability and replication to validate their expression changes by RT-qPCR. 3α-THP up-regulated six genes, two of them were also up-regulated by F. Two genes were up-regulated by P4 alone, however, such an effect was blocked by F when cells were treated with both steroids. The remaining genes were regulated by the combined treatments of 3α-THP + F or P4 + F. An in-silico analysis revealed that promoters of the six up-regulated genes by 3α-THP possess cyclic adenosine monophosphate (cAMP) responsive elements along with CCAAT/Enhancer binding protein alpha (CEBPα) binding sites. These findings suggest that P4 and 3α-THP regulate different sets of genes that participate in the growth of GBMs.

## 1. Introduction

Astrocytomas represent 40–50% of all primary Central Nervous System (CNS) neoplasms and at least 70% of all gliomas. The World Health Organization (WHO) classifies astrocytomas into four grades of malignancy (I–IV) [1]. Grade IV astrocytomas, also known as glioblastoma (GBM), constitute the most common and aggressive brain tumors due to their highly proliferative and infiltrative potential [2]. Steroid hormones such as progesterone (P4), participate in stimulating astrocytomas’ growth [3,4].

Neurons and glial cells metabolize P4, and its metabolites exert numerous actions in the CNS. The main metabolic pathway of P4 comprises two reduction reactions: First, the enzyme 5α-reductase (5αR1/2), which irreversibly reduces the double bond on C4–C5 of P4, metabolizes the hormone to 5α-dihydroprogesterone (5α-DHP). Subsequently, 5α-DHP is reduced by the enzyme 3α-hydroxysteroid dehydrogenase (3αHSD) into allopregnanolone (3α-THP) [5,6].

3α-THP is one of the most extensively studied neurosteroids, given its neuroprotective and myelination effects [7,8], and its role in regulating neural stem cells proliferation [9,10,11]. Regarding its mechanisms of actions, three main pathways have been described: (1) γ-aminobutyric acid receptor A (GABA_A_R) positive modulation [12]; (2) membrane P4 receptors (mPRs) direct activation [13,14]; and (3) pregnane xenobiotic receptor (PXR) interaction. Different reports show that GABA_A_R and mPRs signaling pathways increase cyclic adenosine monophosphate (cAMP) levels thus activating the transcription factor cAMP response binding element protein (CREB) [15], even a crosstalk between both has been suggested [13]. As a ligand-activated transcription factor, PXR induces target gene expression by binding to specific response elements [16].

Recently, we reported that the human GBM cell line U87 expresses 5αR1 and 2. Besides, 3α-THP induces GBM cell proliferation and regulates oncogene expression [17]. Despite these effects, neither the mechanisms of action involved in promoting GBM cell proliferation nor its role in modulating gene expression have been elucidated. Here we report the effects of 3α-THP, P4, and the 5αR inhibitor finasteride (F) on the gene expression profile of U87 cells. Interestingly, both 3α-THP and F induced the expression of genes involved in the maintenance of DNA integrity, DNA replication, and cytoskeleton reorganization.

## 2. Results

### 2.1. 3α-THP and F Promote Gene Expression Changes in U87 Cells

**Figure 1:**
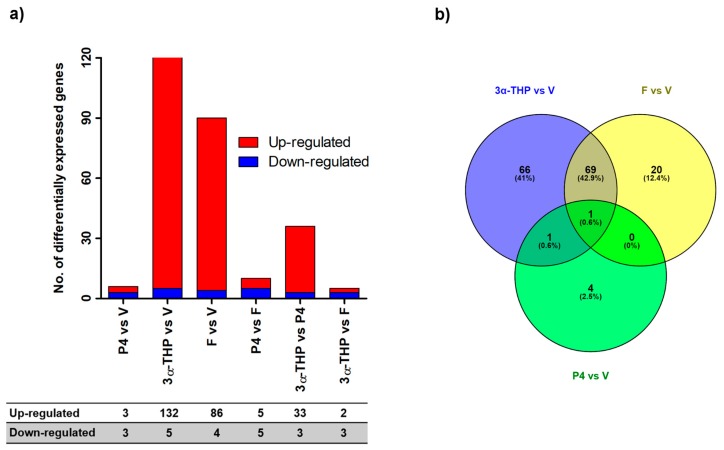
Progesterone (P4), allopregnanolone (3α-THP), and finasteride (F) promote changes in the gene expression profile of U87 cell line. (**a**) The number of differentially expressed genes with a Fc > ±1.5 and *p* < 0.05 between different treatment comparisons. The table shows the number of up- and down-regulated genes. (**b**) The genes that changed their expression under the treatment of P4, 3α-THP, and F vs. vehicle (V), respectively were used to build a Venn’s diagram with the program Venny. 3α-THP and F differentially regulated 69 genes while 3α-THP and P4 regulated one, and the three steroids regulated the expression of one transcript.

Recently, we reported that both 3α-THP and P4 promote U87 cell proliferation after 72 h of treatment [17]. In line with such results, we evaluated the gene expression profile in U87 cells at 72 h of treatment with 3α-THP (10 nM), P4 (10 nM), and F (100 nM). We performed a differential expression analysis by comparing all treatments against the vehicle. The data discussed in this publication have been deposited in NCBI’s Gene Expression Omnibus [18] and are accessible through GEO Series accession number GSE108998 at [19]. Our results show that 3α-THP and F changed the expression of 137 and 90 genes respectively, while P4 modified only six (Figure 1a). Most of the differentially expressed genes were up-regulated by 3α-THP and F (132 and 86, respectively), whereas only five and four genes were down-regulated by each steroid, respectively. P4 up-regulated three genes and down-regulated another three (Figure 1a). Additionally, we performed the comparisons between P4 vs. F, 3α-THP vs. P4, and 3α-THP vs. F. In the first comparison, P4 up- and down-regulated five genes. In the second analysis, 3α-THP up-regulated 33 genes, while in the third comparison it up-regulated two and down-regulated three genes (Figure 1a). Then, we investigated if there were transcripts whose levels were regulated by more than one steroid. Interestingly, 3α-THP and F changed the level of 69 transcripts, while 3α-THP and P4 changed the level of one gene. Furthermore, 66 genes were regulated by 3α-THP alone, 20 by F, and four by P4. One gene was regulated by the three steroids (Figure 1b). The lists of the differentially expressed genes are included in the Tables S1–S6. Principal Component Analysis (PCA) graph of normalized files and heatmaps are in the Supplementary Material (Figure S1). PCA reveals that samples were tightly grouped by steroid treatments except P4, which showed a similar distribution as compared with vehicle (Figure S1a). Heatmaps were performed to determine hierarchical clusters of genes up- or down-regulated by 3α-THP, P4, or F in comparison with vehicle (Figure S1b–d) or by the comparison between steroids (Figure S1e–g). These results are summarized in Figure 1a.

### 2.2. 3α-THP and F Increase the mRNA Levels of Proteins Involved in Several Cellular and Metabolic Processes

**Figure 2:**
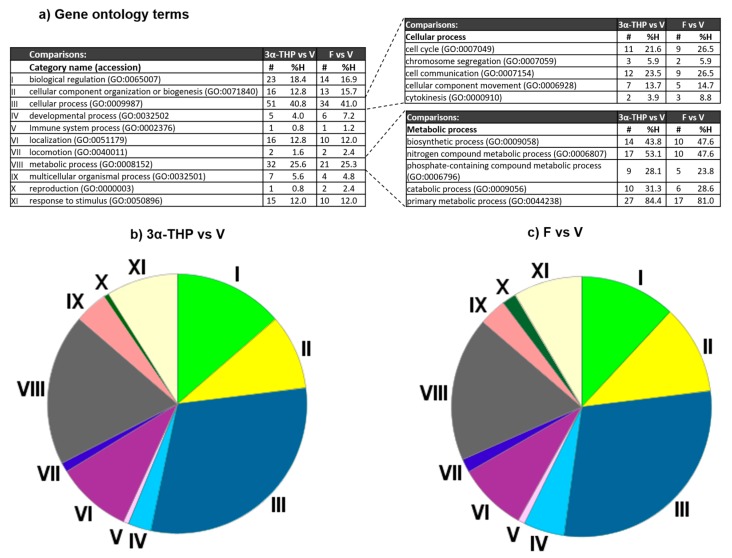
Enrichment analysis of the group of genes regulated by 3α-THP and F. The proteins coded by the differentially expressed genes after 3α-THP and F treatments were analyzed with PANTHER. (**a**) The diverse biological processes (categories) in which the gene products participate are shown in the left table. As the categories of cellular process and metabolic process were ones of the most enriched by 3α-THP and F, we performed a second analysis to determine sub-categories. In the three different tables, the number of genes in each category (column **#**), and the percentage of gene hits against the total number of genes (column %H) for each treatment are indicated. (**b**,**c**) The pie charts show the enriched categories (marked with roman numbers) for the two treatment comparisons.

We performed an enrichment analysis of gene ontology categories using the database PANTHER to identify possible biological processes altered by 3α-THP or F at 72 h of treatment in U87 cells. Cross-examination using the DAVID enrichment algorithm [20,21] confirmed the results. 125 out of the 132 genes up-regulated by 3α-THP treatment were classified under one or more Gene Ontology (GO) categories. The most enriched categories were “cellular process” and “metabolic process”, which included 51 and 32 genes, respectively. Other enriched categories were “biological regulation” (23 genes), “cellular component organization or biogenesis” and “localization” each with 16 genes, and “response to stimulus” (15 genes) (Figure 2a,b). Among the 51 enriched genes in the “cellular process” category, a sub-classification analysis showed that twelve genes code for proteins relevant for cell communication, and eleven for cell cycle processes. The rest of the genes were enriched in the sub-categories “chromosome segregation” (3), “cellular component movement” (7), and “cytokinesis” (2). The analysis for the “metabolic process” category showed that among the 32 enriched genes, 27 were sub-classified into “primary metabolic process”. The remaining genes were classified into the next categories: biosynthetic process (14), nitrogen compound metabolic process (17), phosphate-containing compound metabolic process (9), and catabolic process (10) (Figure 2a).

According to the GO over-representation analysis for F up-regulated genes (Figure 2a,c), the enriched categories were “cellular process”, “biological regulation”, and “metabolic process”, represented by 51, 23, and 32 genes respectively. The analysis of the “cellular process” category showed the enrichment of nine genes in “cell cycle” and in “cell communication” categories, respectively. The remaining genes were sub-classified in “cellular component movement” (7), “cytokinesis” (3), and “chromosome segregation” (2). Moreover, genes grouped in the category of “metabolic process” were sub-classified into the categories: biosynthetic process (10), nitrogen compound metabolic process (10), catabolic process (6), and primary metabolic process (17). These data show that both 3α-THP and F could regulate the same type of genes.

### 2.3. 3α-THP and F Increase the Expression Level of Genes Selected for Validation

Considering the results of both the microarrays and the GO over-representation analyses, we chose ten interesting genes that participate in diverse cell processes for validation (their function is described in Table S7). We selected the genes according to their Fc and *p* values, as well as for their occurrence in the most enriched categories (Table 1). However, among the selected genes, *ESF1* and *RIF1* caught our interest due to their high Fc (*ESF1*: Fc = 3.7, *p* = 0.010, *RIF1*: Fc = 2.69, *p* = 0.015) despite not being included in any PANTHER protein class. Besides, *CCDC91* (Fc = 1.68, *p* = 0.037) was enriched in a Golgi-proteins cluster when the gene enrichment analysis was performed in DAVID, but not in PANTHER.

**Figure 3:**
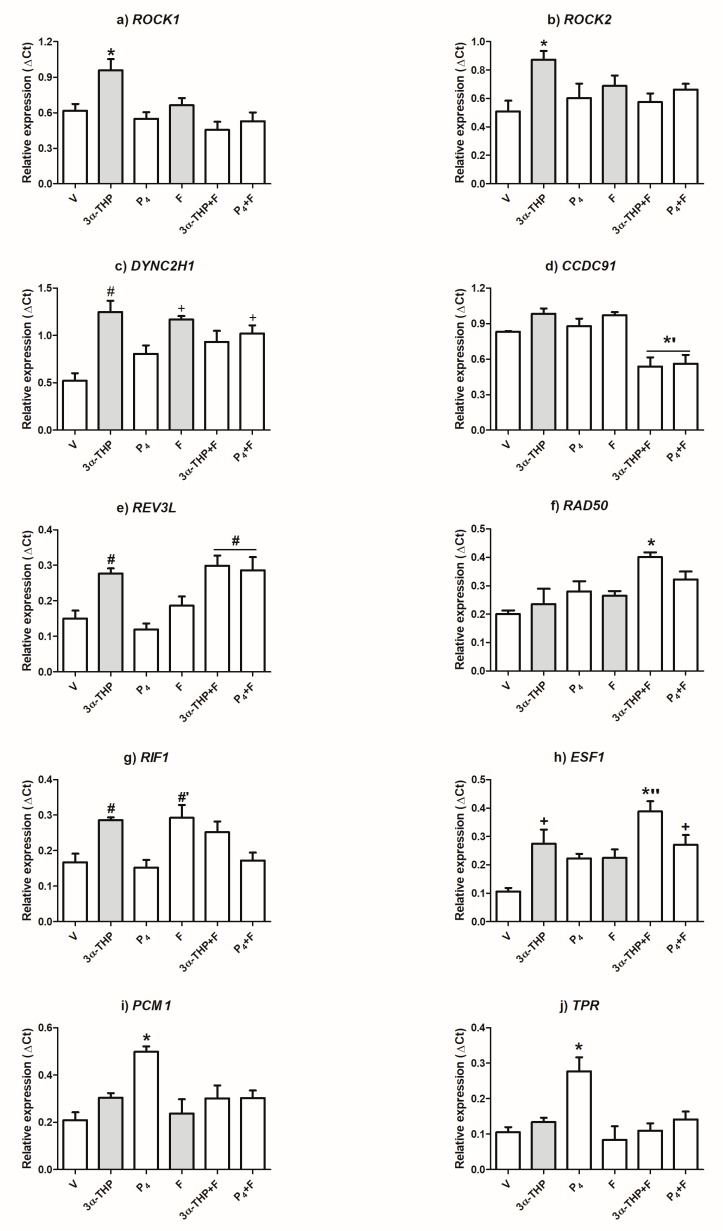
3α-THP, P4, and F regulate the expression of genes selected for validation. RT-qPCR was used to validate ten genes with a Fc > 1.5 in the microarray analysis. U87 cells were treated for 72 h with 3α-THP (10 nM), P4 (10 nM), F (100 nM), 3α-THP + F, and P4 + F. The internal control gene 18S ribosomal RNA was used to calculate the relative expression of each gene according to the Δ*C*t mathematic method. The grey bars represent the microarray results of the up-regulated genes by 3a-THP and/or F. Each column represents the mean ± standard error of mean (SEM), *n* = 3. + *p* < 0.05 vs. V; # *p* < 0.05 vs. V, and P4; #’ *p* < 0.05 vs. V, P4, and P4 + F; * *p* < 0.05 vs. all other treatments; *’ *p* < 0.05 vs. V and single treatments; *’’ *p* < 0.05 vs. V, P4, and F.

For the gene expression validation, we included the treatments used for the microarray analysis (V, 3α-THP, P4, and F) as well as the combined treatments of P4 + F and 3α-THP + F. The latter two were used to determine if 5αR inhibition interfered with the effects of P4 and to discard the fact that F could affect the actions of 3α-THP. The RT-qPCR experiments shown in Figure 3 denote with grey bars the gene expression changes obtained by the microarray analysis. Accordingly, 3α-THP increased the expression of six out of the ten genes chosen for validation, and F changed the expression levels of only one gene (Figure 3).

The microarray experiments showed that ROCK1 and ROCK2 expression increases with 3α-THP and F treatments. Remarkably, the RT-qPCR data indicate that only 3α-THP regulates the expression of these two genes and that F blocked such an effect as shown in the 3α-THP + F treatment (Figure 3a,b). DYNC2H1 expression regulation coincides with the microarray data, and the P4 + F treatment also up-regulated this gene (Figure 3c). Interestingly, the expression level of CCDC91 did not change with any of the single treatments, but it significantly decreased with 3α-THP + F and P4 + F (Figure 3d). The levels of REV3L mRNA augmented with 3α-THP as in the microarray data, and an increase was also observed for both combined treatments (Figure 3e). Concerning RAD50, its expression levels were modified only by 3α-THP + F, but not by the single treatments of 3α-THP and F as expected (Figure 3f). The expression of RIF1 was elevated by 3α-THP according to the microarray results, although F also augmented its expression (Figure 3g). ESF1 was expected to be up-regulated by 3α-THP and F according to the microarray analysis, but the validation showed an increase with the combined treatments (Figure 3h). Lastly, neither 3α-THP nor F modified the expression of PCM1 or TPR (Figure 3i,j, respectively) as in the microarray data. In the case of both genes, P4 promotes an increase in their expression and F significantly abolished this effect as seen in the combined treatment of P4 + F. As 3α-THP up-regulates the expression of six genes chosen for validation, we explored through a bioinformatic analysis the possible mechanism by which 3α-THP could regulate the expression of such genes.

### 2.4. CREB1 and CEBPa Could Mediate 3α-THP-Dependent Transcriptional Effects

**Figure 4:**
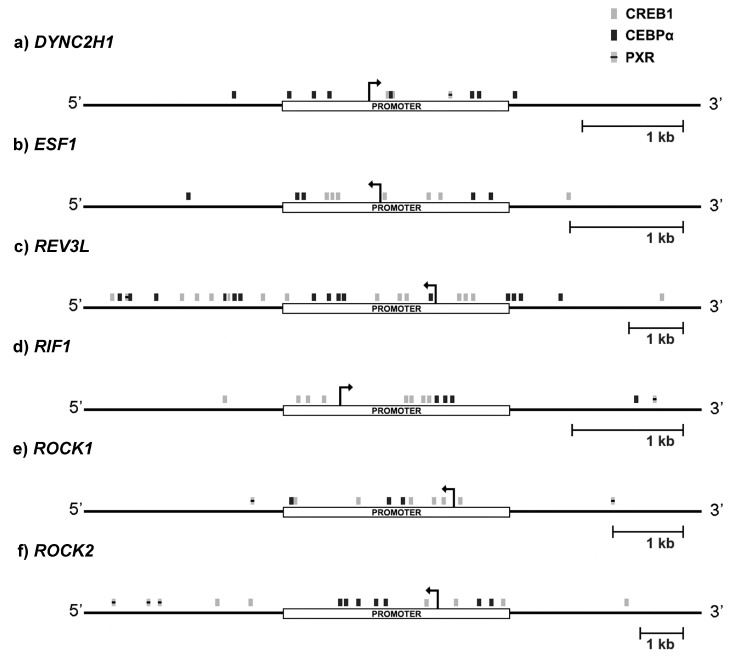
In silico analysis of the regulatory region of the genes up-regulated by 3α-THP. Six of the validated genes were analyzed for their promoter regions and scanned for putative transcription factor binding sites with several bioinformatic tools: JASPAR, UCSC Genome Browser, UGENE, and TRANSFAC. For each gene, a white rectangle indicates the promoter region, and the continuous black lines represent the adjacent regulatory region. Black arrows indicate the transcription start site and the gene transcription direction. The putative binding sites for CREB1 (grey box), CEBPα (black box), and PXR (black lined grey box) are denoted. According to each gene, the scale bar of 1 kb is defined.

As 3α-THP regulates the expression of six out of the ten genes chosen for validation, we performed an in-silico analysis for putative transcription factor binding sites (TFBS) in their promoter regions. The bioinformatic analysis focused on the transcription factors CCAAT/Enhancer binding protein alpha (CEBPα), CREB1, and PXR, which are known to participate in 3α-THP mechanisms of action. Four bioinformatic tools were used for the prediction of the TFBS: JASPAR, Unipro UGENE v.1.26.3, UCSC Genome Browser, and TRANSFAC. UGENE software was used to compile the data. Only the binding sites predicted by two or more programs (similarity score > 0.8, *p* < 0.05) were considered as positive hits. The selected genes for this analysis were DYNC2H1 (Figure 4a), ESF1 (Figure 4b), REV3L (Figure 4c), RIF (Figure 4d), ROCK1 (Figure 4e), and ROCK2 (Figure 4f). In every analyzed gene regulatory region, the most abundant TFBS were for CREB1 and CEBPα and few for PXR. Except for REV3L, most of the binding sites are located in the promoter regions. These data suggest that these factors should regulate the 3α-THP-dependent transcription of the selected genes.

## 3. Discussion

This study aimed to evaluate the gene expression profile changes produced by 3α-THP, P4, and F in U87 cells, and correlate them with our previous proliferation data [17]. First, we performed a PCA of the microarray data after their normalization. Displaying the principal components of the microarray data allows possible batch effects to be identified, such as technical variables affecting the interesting biological variability between conditions [22], in this case, 3α-THP, P4, and F treatments. This analysis let us identify some additional changes in gene expression more precisely related to 3α-THP and F treatments, than with the simple normalization without determining principal components. We found a tight grouping between treatments, except for P4 and V. This correlates with the fact that P4 and vehicle treatments display a very similar expression profile, since P4 altered the expression of only six genes as compared with vehicle. Moreover, the microarray analysis showed that 3α-THP promotes more changes in the gene expression profile of U87 cells than P4. This difference could be due to their specific mechanisms of action. There are two main mechanisms of action of P4: the classical and the non-classical. The first one depends on the binding of P4 to its intracellular receptor (PR) that functions as a transcription factor, and the second one is mediated by mPRs, which are G protein-coupled receptors that activate different signaling pathways involving the production of second messengers (for review see [23]). We and others have reported that P4 induces the expression of cyclins, growth factors such as vascular endothelial growth factor (VEGF) or epidermal growth factor (EGF), and receptors like EGF(R) through PR activation [24]. However, these effects had been detected at 12 h or less of treatment [24,25]. The few changes in gene expression with P4 at 72 h of treatment could be associated with its mechanism of action: once PR is active, the ligand-dependent phosphorylation of the receptor that increases its transcriptional activity [26,27] also promotes its degradation by de 26S proteasome. PR degradation has been reported to occur between 4 and 12 h in breast cancer cells [28], whereas in the human astrocytoma cell line U373 takes 3–5 h [26]. Nevertheless, we have previously reported that 3α-THP promotes U87 cell proliferation in a very similar manner as P4 at 72 h of treatment [17], despite not presenting affinity to PR [29].

Another interesting observation of our microarrays results is that groups of 3α-THP and F are more related in the PCA analysis. According to this, we found many regulated-genes by both F and 3α-THP, than those discovered with a simple normalization and gene expression analysis. F regulates 69 genes also modified by 3α-THP. When the gene ontology analysis of up-regulated genes by 3α-THP or F was performed, we found a higher enrichment in the category of “cellular process”, specifically in the subcategories of “cell cycle”, “cell communication” and “cell component movement”. The category of “metabolic process” was highly enriched in all the subcategories reported. Despite F being a well-known 5αR inhibitor, it also modifies the expression of a wide range of genes in different biological systems [30,31,32]. However, the mechanisms by which F exerts its agonistic effects are not fully elucidated. Wu (2013) and coworkers proposed that, due to their steroid-based structure, F could interact with the androgen receptor (AR) and modulate the expression of target genes such as the prostate-specific antigen in prostate cancer cell lines. Nevertheless, these effects might depend on the inherent cellular characteristics. Another hypothesis suggests that F and/or other 5αR inhibitors with steroid structure could interfere with the formation of the active complex of the AR and its natural ligand, dihydrotestosterone [33,34]. Besides, F could modify the levels of other steroids susceptible to 5α-reduction including testosterone, androstenedione, aldosterone, cortisol, and deoxycorticosterone. These steroids modulate gene expression, and their effects could be affected by the F treatment [35]. Additionally, there are other P4 metabolites whose synthesis does not directly depend on 5αR. For example, there is 3α-hydroxy-4-pregnen-20-one (3αHP), which is the product of the direct reduction of P4 by the enzyme 3αHSD. This metabolite has a similar mechanism of action as 3α-THP [36], and it is also a positive modulator of GABA_A_R [37]. Therefore, F treatment could enhance alternative P4 metabolic pathways as described. Despite the fact that F did not increase proliferation in U87 GBM cell line in our previous work [17], here we suggest that F and 3α-THP should enhance the malignancy of the cells since the genes regulated by these steroids are related with cell migration, DNA repair and cell cycle.

Particularly interesting is the 3α-THP-dependent regulation of *ROCK1* and *ROCK2* expression, which are key regulators of cell morphology, cell invasion, migration, and proliferation [38,39,40]. In fact, Rho/ROCK is one of the most important pathways that favors GBM cell migration, as ROCK phosphorylation targets include essential proteins involved in actomyosin contraction [41,42]. Recently, reports show that 3α-THP promotes rat Schwann cell migration in culture [43], suggesting that the induction of ROCK expression should increase GBM cell migration.

Remarkably, both 3α-THP and F regulated RIF1, indicating that other metabolites besides 3α-THP, whose levels might be modified by the treatment of F, could be responsible for this increase. Besides, the promoter of this gene presented many CREB1 binding sites. CREB acts downstream of the signaling pathway of GABA_A_R, a receptor targeted by 3α-THP and other P4 metabolites. RIF1 is of particular interest, given its relevance for the maintenance of the DNA stability and induction of DNA replication [44,45].

In contrast, 3α-THP, F, and the combined treatment of P4 + F regulate DYNC2H1 expression, suggesting different mechanisms of action promoted by other P4 metabolites such as the ones synthesized by 3αHSD. The cytoplasmic localization of DYNC2H1 protein has been associated with resistance to the primary GBM chemotherapeutic agent temozolomide [46].

Furthermore, *REV3L* and *ESF1* were regulated by 3α-THP and by the two combined treatments, suggesting that the effect of 3α-THP is maintained even with F treatment. Besides, 5αR-independent P4 metabolism could contribute to the up-regulation of such genes when treated with P4 + F. Interestingly, at least one of the combined treatments induced the expression of genes such as *CCDC91* and *RAD50*, and thus, they might be regulated differently. In the case of *TPR* and *PCM1*, both involved in cell division, P4 alone increased the expression of both genes, and its effect was blocked by F as observed in the combined treatment. This result also indicates that the P4-dependent expression of *PCM1* and *TPR* could be mediated by other 5α-reduced metabolites such as 5α-DHP, which can directly activate PR [29].

As mentioned before, reports indicate that 3α-THP could bind to specific mPRs. These receptors comprise the class II of the progesterone and adipoQ receptor family (PAQR) which includes five members: PAQR7 (mPRα), PAQR8 (mPRβ), PAQR5 (mPRγ), PAQR6 (mPRδ), and PAQR9 (mPRε). It has been suggested that the first three members are coupled to inhibitory G-proteins (G_i_), whereas the lasts two activate stimulatory G-proteins (G_s_) (for review see [47]). The high affinity of 3α-THP for mPRδ [14] could lead to the activation of the adenylyl cyclase, followed by an increase of intracellular cAMP levels, activation of the protein kinase A (PKA), and phosphorylation of CREB transcription factor. In fact, Shimizu and coworkers (2015) reported that H-89, a PKA inhibitor, blocked the effect of 3α-THP on the drebrin clusters density, an important protein in the formation of dendritic spines [15]. The prediction of a high number of CREB binding sites in the promoter region of the validated genes suggests that 3α-THP promote gene expression in a CREB-dependent manner in GBM. Besides, CREB expression and activation is directly related with the astrocytoma grade [48] and participates in the up-regulation of genes involved in DNA repair such as RAD50 as well as genes promoting cell proliferation and cytokinesis in PC12 cells [49]. Interestingly, 3α-THP (10 nM) induces CREB phosphorylation in rat Schwann cells [50]. Also, CREB binding sites are commonly found near CEBPα and CEBPβ sites, and both transcription factors are required to obtain a robust expression of different genes [51,52]. In U87 cells, there is evidence of a high CEBPα expression [53], which led us to determine the presence of CEBPα sites along with CREB sites in the promoter of the validated genes.

Regarding PXR, we cannot discard the possibility that this receptor regulates the expression of the evaluated genes, given that 3α-THP can directly activate it in vivo [16]. PXR modulates the expression of genes whose products are mainly involved in xenobiotic metabolism [54].

This work shows the importance of determining 3α-THP mechanisms of action in GBMs, and to take into consideration steroid hormone metabolism when studying its effects. Besides, it opens new questions regarding the activation of CREB and its participation along with CEBP in regulating the expression of the validated genes. Nevertheless, it is crucial to determine the role of the studied genes in the molecular and cellular biology of GBMs while considering physiological P4 levels and their metabolites, since they could participate in cancer progression.

## 4. Materials and Methods

### 4.1. Cell Culture and Treatments

U87 human glioblastoma cell line (purchased from ATCC, Georgetown, WA, USA) was cultured in phenol red and high glucose Dulbecco’s Modified Eagle’s medium (DMEM, In vitro, Mexico City, Mexico) supplemented with 10% fetal bovine serum (FBS), 1 mM pyruvate, 2 mM glutamine, and 0.1 mM non-essential amino acids, at 37 °C in a humidified 5% CO_2_ atmosphere. 2 × 10^5^ cells were plated in 12-well plates for the microarray experiments. 8 × 10^4^ cells were plated in 6-well plates to validate the microarray data. 24 h before steroids treatments, the medium was changed for phenol red-free and high glucose DMEM (In vitro, Mexico City, Mexico), supplemented with charcoal-stripped FBS. Treatments for microarray analysis were: vehicle (V, 0.1% DMSO), P4 (10 nM), 3α-THP (10 nM), F (100 nM). Treatments for gene expression validation by RT-qPCR were: (V, 0.1% DMSO), P4 (10 nM), 3α-THP (10 nM), F (100 nM), P4 + F and 3α-THP + F (same concentrations) for 72 h; all hormones were purchased from Sigma Aldrich (St. Louis, MO, USA).

### 4.2. RNA Extraction and Microarrays

After 72 h of treatment, total RNA was extracted by the phenol-guanidine isothiocyanate-chloroform method using TRI Reagent (Molecular Research Center Inc., Cincinnati, OH, USA) according to the manufacturer’s recommendations. RNA concentration and purity were determined with the NanoDrop 2000 Spectrophotometer (Thermo Fisher Scientific, Waltham, MA, USA). RNA integrity was assessed on the Agilent 2100 Bioanalyzer (Agilent Technologies, Santa Clara, CA, USA) and the software 2100 Expert. Samples with an RNA integrity number (RIN) value above 8.0 were used for further processing. Microarray experiments were performed in the Microarray Core Facility at the National Institute of Genomic Medicine (INMEGEN, Mexico City, Mexico). The WT cDNA Synthesis and Amplification kit (Thermo Fisher Scientific, Waltham, MA, USA) was used to obtain the double strand cDNA, while the cRNA was obtained by in vitro transcription. The hybridization was performed with the GeneChipTM Human Gene 1.0 ST arrays (Thermo Scientific, Waltham, MA, USA) according to the standard protocol, and exogenous controls were included.

Raw microarray intensity data were pre-processed and quantile normalized using the Transcriptomic Analysis Console (TAC) Software 4.0.1. (Thermo Fisher Scientific). The principal component analysis of the normalized data is shown in Figure S1. We performed an unpaired one-way ANOVA and a false discovery rate (FDR) analysis. For further confirmation, we used different Bioconductor packages in the statistical platform R. We first employed a robust multiarray analysis (RMA) to transform and normalize the data into log2 data. Then, differential expression analysis with the Linear Models of Microarray Data (LIMMA) package was performed using the moderated *t*-test and the Benjamini-Hochberg FDR as statistical analyses. Here, we report the results obtained with the TAC Software 4.0.1. Differential gene expression with a fold change (Fc) <−1.5 or >1.5 and *p* < 0.05 was considered as statistically significant and non-random. After the normalization and statistical analysis of the raw microarray data, all steroid treatments were compared against the vehicle (V, 0.1% DMSO).

The lists of differentially expressed genes among conditions were then exported to Venny 2.1.0 [55], and then to the Protein Analysis Through Evolutionary Relationships (PANTHER) database [56,57,58] (available at [59]) to determine the biological processes enrichment. With the Database for Annotation, Visualization and Integrated Discovery (DAVID) v6.8 [20,21], available at [60], the ontology results were also confirmed.

### 4.3. Validation of Selected Differentially Expressed Genes by RT-qPCR 

The genes with the highest Fc among treatment comparisons and with relevance in the enriched biological processes were selected. To validate the expression of the chosen genes, we designed oligonucleotides in the primer-BLAST tool from the National Center for Biotechnology Information (NCBI) database available at [61]. The oligonucleotide sequences and the amplicon lengths are shown in Table 2.

Total RNA was extracted after 72 h of treatment with vehicle, P4, 3α-THP, F, P4 + F, and 3α-THP + F as described. The concentration and purity of RNA were determined using the NanoDrop, as well as its integrity in a 1.5% agarose gel electrophoresis. cDNA was synthesized from 1 μg of total RNA using the M-MVL reverse transcriptase according to the manufacturer’s instructions (Invitrogen, Carlsbad, CA, USA) with oligo-dT_12–18_ as primers. qPCR was performed in a LightCycler 1.5 using the FastStart DNA Master SYBR Green I reagent (Roche Diagnostics, Mannheim, Germany) according to the manufacturer’s protocol. Briefly, 1 μg of cDNA of the previous reaction was used to perform qPCR, and each gene was amplified (Table 2) along with the endogenous reference gene *18S* ribosomal RNA to quantify the relative expression by the Δ*C*t method [62,63]. Duplicate samples for each of the three independent experiments were included.

The data were analyzed and plotted in the GraphPad Prism 5 software for Windows XP (GraphPad Software, Version 5.01, La Jolla, CA, USA). The statistical analysis of the relative gene expression levels was one-way ANOVA followed by a Tukey post-hoc test. Values of *p* < 0.05 were considered statistically significant.

### 4.4. Bioinformatic Analysis of Transcription Factor Binding Sites

The putative transcription factor binding sites (TFBS) analysis for the selected genes was performed using several bioinformatic tools. First, promoters and gene sequences were obtained from the NCBI database [64]. Then, the promoter regions and transcription start site (TSS) were determined with the Ensembl database [65] and confirmed by the Eukaryotic Promoter Database (EPD) [66]. We searched for putative binding sites for CREB1, CEBPα and PXR using JASPAR [67], UCSC Genome Browser [68], Unipro UGENE v.1.26.3 software [69], and TRANSFAC software [70]. For all analyzed genes, we established as potential TFBS the ones predicted by two or more databases with a matrix similarity score greater than 0.8 and a value of *p* < 0.05.

## 5. Conclusions

In this work, we show that 3α-THP promotes the expression of genes involved in DNA stability maintenance and replication, in the reorganization of the cytoskeleton, and the transport of different cargo compounds in U87 GBM cell line. Besides, F blocked the effects of P4 on the expression of genes involved in cell division (TPR and PCM1), suggesting that the inhibition of 5αR should affect GBM progression. Further investigation is necessary to determine whether F could enhance the malignancy of GMB cells since many genes related to this process were regulated by both 3α-THP and F.

## Figures and Tables

**Table 1:** The microarray data (Fc and *p*-value) and the ontogeny analysis of the selected genes for validation.

Gene Full Name	Treatment vs. V (Fc)		Treatment vs. V (*p*-Value)		Ontogeny Categories
	3α-THP	F	3α-THP	F	
ESF1 nucleolar pre-rRNA processing protein homolog (*ESF1*)	3.07	2.01	0.0103	0.044	ND
Translocated promoter region, nuclear basket protein (*TPR*)	2.75	-	0.010	-	Biological regulation Cellular component organization or biogenesis Cellular process Localization
Replication timing regulatory factor 1 (*RIF1*)	2.69	-	0.015	-	ND
RAD50 double-strand break repair protein (*RAD50*)	2.31	2.16	0.010	0.022	Biological regulation Cellular component organization or biogenesis Cellular process Metabolic process Reproduction Response to stimulus
Rho-associated coiled-coil containing protein kinase 1 (*ROCK1*)	1.94	2.04	0.009	0.018	Cellular process Developmental process Localization
Rho-associated coiled-coil containing protein kinase 2 (*ROCK2*)	1.94	1.8	0.041	0.034	Cellular process Developmental process localization
REV3-like, DNA directed polymerase zeta catalytic subunit (*REV3L*)	1.86	-	0.014	-	Cellular process Metabolic process
Pericentriolar material 1 protein (*PCM1*)	1.68	1.54	0.037	0.044	Biological regulation Cellular component organization or biogenesis Cellular process Localization
Dynein cytoplasmic 2 heavy chain 1 (*DYNC2H1*)	1.64	1.55	0.001	0.023	Cellular process Localization
Coiled-coil domain containing 91, P56 protein (*CCDC91*)	1.63	-	0.04	-	ND

Note: ND = not determined; - = no changes in gene expression under this comparison.

**Table 2:** Designed primers used for different gene amplifications.

Gene	Primer Sequence 5′→3′	Amplified Fragment (bp)
*ESF1*	FW: GCTCCTCGTGCTGATGAGATTA	176
	RV: TGCTCTTCCTTCATCCTCTCCT	
*PCM1*	FW: TCAAGACAAGAAAAGCGTCTGC	180
	RV: GGGCTGAATGTCTGTTCCTACT	
*TPR*	FW: TTTGGCACAGTTTCGGCTAC	164
	RV: TCTTCCTCAGTTCCTACAGGTG	
*RIF1*	FW: TAATAAGGTTCGCCGTGTCTCC	177
	RV: CCTTTGGCTGAAGTGGTATTATGC	
*REV3L*	FW: TGAGAAATGAGGTGGCTCTAAC	168
	RV: CACGGACACGGCTAACATAA	
*RAD50*	FW: GCCTCACTCATCATTCGCCT	168
	RV: AAGCTGGAAGTTACGCTGCT	
*ROCK1*	FW: ATGGAACCAGTACAACAAGCTGA	159
	RV: GCATCTTCGACACTCTAGGGC	
*ROCK2*	FW: GAAGAGCAGCAGAAGTGGGT	170
	RV: GGCAGTTAGCTAGGTTTGTTTGG	
*CCDC91*	FW: AAGTCAGGAAACTGTTAAGGCAG	152
	RV: ACAGGCTTCTTTGGCGGAT	
*DYNC2H1*	FW: GCTTGGCGGAGCAGATTAAA	159
	RV: CCAGGATGCCCGATTCAGTAT	
*18S*	FW: AGTGAAACTGCAATGGCTC	167
	RV: CTGACCGGGTTGGTTTTGAT	

Note: FW = forward primer; RV = reverse primer. Official full names of the genes are shown in Table 1.

## Supplementary Materials

Supplementary materials can be found at www.mdpi.com/1422-0067/19/3/864/s1.
