# Supplementary material for: Allopregnanolone Alters the Gene Expression Profile of Human Glioblastoma Cells
Source: Int J Mol Sci. 2018 Mar 15;19(3):864. doi: 10.3390/ijms19030864 (PMC5877725; doi:10.3390/ijms19030864)
Supplement: Supplementary file 1 [file ijms-19-00864-s001.pdf]

# Supplementary Materials

## Supplementary Figure 1

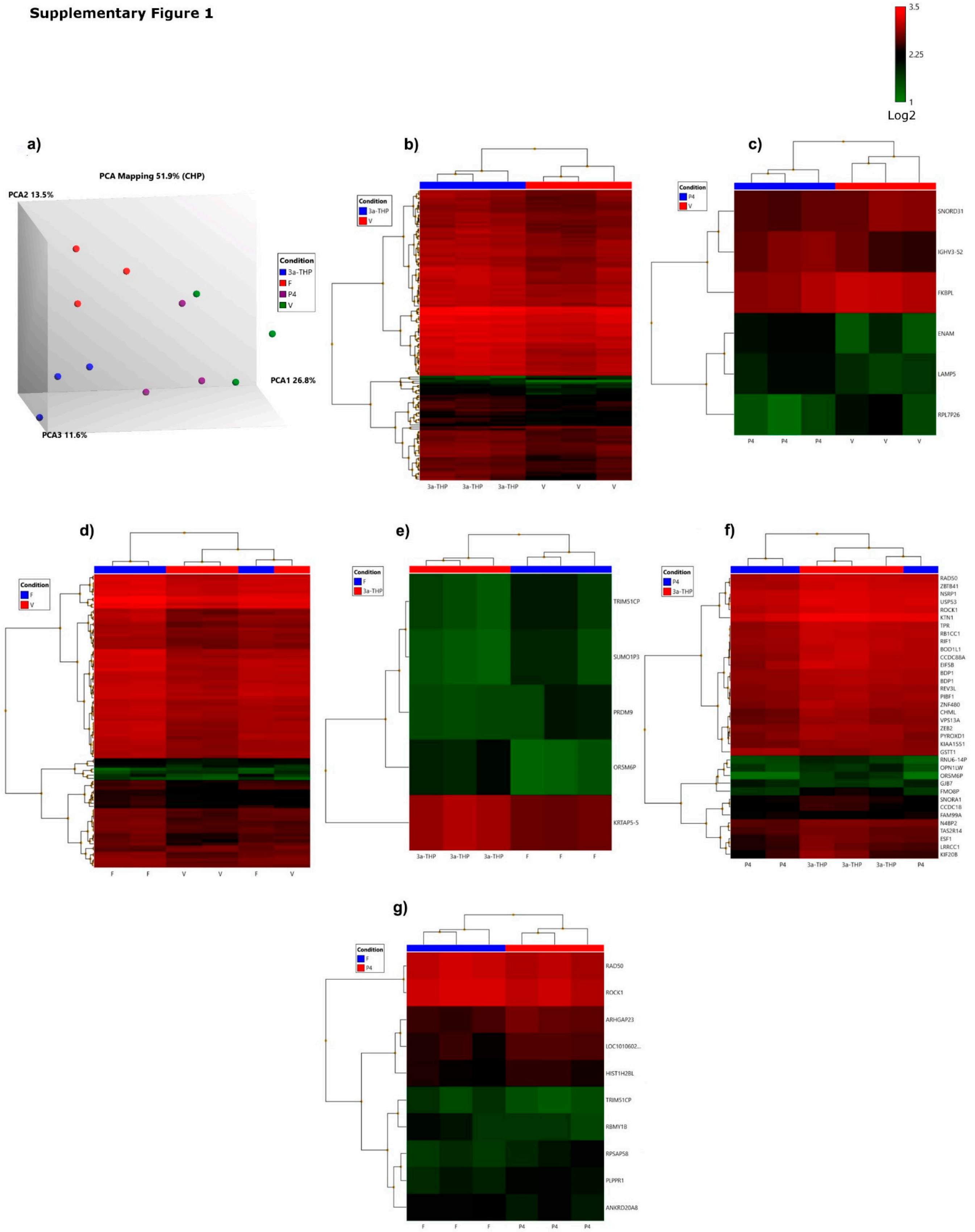

**Figure S1.** Principal component analysis of microarray data and heat maps of the different comparisons between treatments. **a**) The Principal component analysis shows a tight grouping between treatments: allopregnanolone (3α-THP, blue dots), finasteride (F, red dots), progesterone (P4, purple dots), Vehicle (V, DMSO 0.1 %, green dots). Each axis represents the principal components of the microarray data (PCA1, PCA2, and PCA3), and the variance along each component is indicated; (b–g) Heat maps display the clusters of differentially expressed genes (Fc <-1.5 or >1.5; o p <0.05) between treatments comparisons: **b**) 3α-THP vs V; **c**) P4 vs V; **d**) F vs V; **e**)3α-THP vs F; **f**) 3α-THP vs P4; **g**) P4 vs F. The magnitude of expression of each gene was log2 transformed and represented as indicated in the color scale. Each row represents a gene, and each column represents each sample.

In this section, we show the tables of the differential expressed genes when compared 3α-THP, P4, and F with respect to V, or as a result of the comparisons between treatments. We show results only of annotated genes.

**Table S1.** Differential gene expression profile of U87 cells treated with 3α-THP vs V.

| **3α-THP vs V** | | | | | | | | |
|---|---|---|---|---|---|---|---|---|
| **Gene Symbol** | **Fc** | **P-val** | **Gene Symbol** | **Fc** | **P-val** | **Gene Symbol** | **Fc** | **P-val** |
| OR5M6P | 2.26 | 5.28E-06 | ROCK2 | 1.94 | 0.0102 | PHF3 | 2.19 | 0.0218 |
| RALGAPA1 | 1.52 | 0.0004 | THOC2 | 1.52 | 0.0104 | ATRX | 2.34 | 0.0222 |
| DLEU2 | 1.61 | 0.0004 | BST2 | -1.63 | 0.0105 | ZNF292 | 1.82 | 0.0223 |
| ARID4B | 1.62 | 0.0006 | BDP1 | 1.72 | 0.0105 | NOL8 | 1.51 | 0.0227 |
| ZNF107 | 1.6 | 0.0008 | BDP1 | 1.71 | 0.011 | MAP9 | 2.09 | 0.0235 |
| CLEC2D | 1.54 | 0.0019 | OR4F14P | 1.54 | 0.011 | CCDC82 | 2.09 | 0.0237 |
| LOC105374874 | 1.97 | 0.002 | EIF5B | 2.85 | 0.0111 | CEP135 | 1.71 | 0.0246 |
| SCAF11 | 1.53 | 0.0024 | CHML | 2.37 | 0.0115 | ERV3-1; ZNF117 | 1.77 | 0.0248 |
| RAD50 | 2.31 | 0.0031 | OCR1 | 1.69 | 0.0116 | CEP295 | 1.71 | 0.0249 |
| KIAA2026 | 1.6 | 0.0031 | ESCO1 | 1.63 | 0.0116 | ETAA1 | 1.66 | 0.0249 |
| TAS2R50 | 1.71 | 0.0032 | NBEAL1 | 1.51 | 0.0116 | CNTLN | 1.64 | 0.0253 |
| ESF1 | 3.07 | 0.0032 | USP53 | 1.82 | 0.0119 | SMC4 | 2.24 | 0.0254 |
| SNORA1; TAF1D; SNORA8 | 1.89 | 0.0032 | SMC5 | 1.53 | 0.012 | GOLGA4 | 1.93 | 0.0254 |
| ZEB2 | 1.63 | 0.0038 | EXOC5 | 1.55 | 0.0125 | SMC3 | 1.67 | 0.0261 |
| ROCK1 | 1.94 | 0.004 | ZNF480 | 2.15 | 0.0126 | RBMY1B; RBMY1D; RBMY1E | 1.74 | 0.0267 |
| RBM41 | 1.82 | 0.0044 | GCC2 | 1.94 | 0.0131 | DST | 2 | 0.0274 |
| DYNC2H1 | 1.64 | 0.0046 | OR4F29; OR4F3; OR4F16; OR4F21 | 1.52 | 0.0132 | RBBP6 | 1.59 | 0.0277 |
| REV3L | 1.86 | 0.0049 | BAZ2B | 1.54 | 0.0132 | CEP152 | 1.98 | 0.0278 |
| ZNF267 | 1.97 | 0.0049 | SLK | 1.77 | 0.0133 | TRNT1 | 1.51 | 0.028 |
| CEP112 | 1.5 | 0.005 | CWC22 | 1.51 | 0.014 | TRIP11; ATXN3 | 2.16 | 0.0285 |
| PPIG | 2.05 | 0.0051 | SAMD9 | 2.7 | 0.014 | APC | 1.87 | 0.0299 |
| NEMF | 1.92 | 0.0051 | VPS13A | 2.15 | 0.0145 | GOLGB1 | 1.77 | 0.0309 |
| RB1CC1 | 2.46 | 0.0054 | JMJD1C | 1.85 | 0.0145 | PCGF5 | 1.58 | 0.0312 |
| PIBF1 | 1.69 | 0.0054 | MMP16 | 1.61 | 0.0147 | ARID4A | 1.7 | 0.0312 |

| | | | | | | | | |
|---|---|---|---|---|---|---|---|---|
| RIF1 | 2.69 | 0.0055 | RGPD1; RGPD6; RGPD5; RGPD4; RGPD8; RGPD2 | 1.5 | 0.0148 | ZNF644 | 1.62 | 0.0324 |
| MANEA | 1.52 | 0.0057 | TAS2R19; TAS2R30 | 1.99 | 0.0151 | OR4F15 | 1.6 | 0.0328 |
| KIAA1551 | 1.57 | 0.006 | CCDC18 | 1.7 | 0.0151 | PHIP | 1.7 | 0.0329 |
| NSRP1 | 1.62 | 0.0063 | VPS13C | 1.7 | 0.0154 | KIF20B | 3.25 | 0.0346 |
| TPR | 2.75 | 0.0063 | RBM7 | -1.58 | 0.0158 | RPL7P26 | -1.77 | 0.0366 |
| CCDC88A | 2.97 | 0.0064 | UACA | 1.68 | 0.0159 | HNRNPU | 1.69 | 0.0372 |
| ZNF638 | 1.56 | 0.0067 | NIPBL | 2.25 | 0.0167 | FMO8P | 1.56 | 0.038 |
| CCDC186; MIR2110 | 2.48 | 0.0067 | RNU6-79P | 1.68 | 0.0169 | DCUN1D1 | 1.53 | 0.0383 |
| ODF2L | 1.54 | 0.0068 | ATRX | 1.91 | 0.0172 | HEXA-AS1 | 1.51 | 0.0391 |
| LRRCC1 | 2.17 | 0.007 | MIS18BP1 | 2.95 | 0.0173 | RANBP2 | 1.87 | 0.0405 |
| ZC3H13 | 1.94 | 0.0072 | ANKRD30B | 1.53 | 0.0177 | ANKRD36B | 1.56 | 0.0405 |
| TAS2R31 | 2.32 | 0.0074 | ENAM | 1.67 | 0.0183 | SMC2 | 2.27 | 0.0406 |
| FAM216B | 1.62 | 0.0078 | EEA1 | 2.42 | 0.0187 | MIR186 | 1.87 | 0.0421 |
| MNS1 | 2.46 | 0.008 | ACAA1 | -1.64 | 0.0188 | ANKRD36; ANKRD36B | 1.57 | 0.0423 |
| FAM99A | -1.61 | 0.0081 | IFT74 | 1.71 | 0.0192 | CHD1 | 1.9 | 0.0433 |
| PCM1 | 1.68 | 0.0082 | PIK3C2A | 1.71 | 0.0194 | NPAT | 1.56 | 0.0435 |
| KTN1 | 2.68 | 0.0086 | CASP8AP2 | 2.18 | 0.0195 | PRO2012 | 2.02 | 0.0441 |
| TAS2R14 | 1.85 | 0.0086 | ANKRD36 | 1.61 | 0.0198 | SMC6 | 1.79 | 0.0449 |
| BOD1L1 | 2.22 | 0.0095 | KIF18A | 2.52 | 0.0201 | ZNF518A | 1.63 | 0.0451 |
| ZBTB41 | 1.95 | 0.0095 | ANKRD36; ANKRD36C | 1.53 | 0.0209 | TAS2R13 | 1.53 | 0.0454 |
| N4BP2 | 1.9 | 0.0097 | LPAR4 | 1.54 | 0.0212 | SLC16A7 | 1.53 | 0.0486 |
| HMGN5 | 1.64 | 0.0099 | CEBPZ | 1.54 | 0.0216 | FAR1 | 1.51 | 0.0489 |
| | | | | | | KCNT2 | 1.63 | 0.0499 |

Note: Fc = Fold change

**Table S2.** Differential gene expression profile of U87 cells treated with P4 vs V.

| P4 vs V | | | | | | | | |
|---|---|---|---|---|---|---|---|---|
| **Gene Symbol** | **Fc** | **P-val** | **Gene Symbol** | **Fc** | **P-val** | **Gene Symbol** | **Fc** | **P-val** |
| ENAM | 2.27 | 0.0015 | FKBPL | -1.5 | 0.0131 | SNORD31; SNHG1 | -1.51 | 0.0189 |
| LAMP5 | 1.53 | 0.0041 | FKBPL | -1.5 | 0.0131 | IGHV3-52; IGHV3-33; IGHV3-35; IGHV3-38 | 1.65 | 0.0289 |
| | | | | | | RPL7P26 | -1.9 | 0.0448 |

**Table S3.** Differential gene expression profile of U87 cells treated with F vs V.

| F vs V | | | | | | | | |
|---|---|---|---|---|---|---|---|---|
| Gene Symbol | Fc | P-val | Gene Symbol | Fc | P-val | Gene Symbol | Fc | P-val |
| BIVM | -1.53 | 0.0027 | NEMF | 1.73 | 0.0168 | PIK3C2A | 1.63 | 0.029 |
| CEP112 | 1.72 | 0.0029 | GCC2 | 2.74 | 0.0171 | ZBTB20 | 1.66 | 0.0291 |
| SCAF11 | 1.53 | 0.0044 | KTN1 | 2.55 | 0.0177 | ANKRD36; ANKRD36C | 1.72 | 0.0299 |
| CEP162 | 1.62 | 0.005 | LRRCC1 | 1.95 | 0.0186 | OR7E121P | -1.63 | 0.0307 |
| ANKRD36 | 1.81 | 0.0055 | SLK | 1.8 | 0.0196 | VPS13C | 1.64 | 0.0312 |
| RNU5A-1 | -1.52 | 0.0057 | TPR | 2.59 | 0.02 | LOC100287934 | 1.6 | 0.0327 |
| RAD50 | 2.16 | 0.0061 | PCM1 | 1.54 | 0.0204 | ZNF292 | 2.13 | 0.0333 |
| ZNF37A | 1.58 | 0.0065 | ZEB2 | 1.53 | 0.0204 | MAP9 | 2.15 | 0.0338 |
| ZNF721; ABCA11P | 1.67 | 0.0066 | CCDC88A | 2.86 | 0.0205 | TMF1 | 1.59 | 0.0339 |
| RBMY1B; RBMY1D; RBMY1E | 2.01 | 0.0066 | NBEAL1 | 1.55 | 0.0205 | ENAM | 1.66 | 0.0349 |
| MANEA | 1.52 | 0.0071 | ANKRD30 B | 1.7 | 0.021 | CEP135 | 1.58 | 0.0353 |
| TRIM51CP | 1.72 | 0.0074 | TEX9 | 1.67 | 0.0214 | CASP8AP2 | 2.14 | 0.0361 |
| RBM41 | 1.9 | 0.0075 | ADAM28 | 1.51 | 0.0215 | ATRX | 2.37 | 0.0364 |
| ROCK1 | 2.04 | 0.008 | CHD9 | 1.57 | 0.0221 | EIF5B | 2.23 | 0.0367 |
| SNORA1; TAF1D; SNORA8 | 1.57 | 0.0085 | RIF1 | 2.34 | 0.0223 | RBBP6 | 1.56 | 0.0384 |
| KIAA2026 | 1.56 | 0.0087 | ESCO1 | 1.78 | 0.0225 | OCR1 | 1.54 | 0.0394 |
| DYNC2H1 | 1.55 | 0.0091 | ZNF267 | 1.67 | 0.0227 | LOC100287934; LOC100287497 | 1.61 | 0.0396 |
| CCDC186; MIR2110 | 2.83 | 0.0097 | ZC3H13 | 1.74 | 0.023 | ATRX | 2.91 | 0.0396 |
| ANKRD20A8P; ANKRD20A2 | 1.53 | 0.0099 | SAMD9 | 3.07 | 0.0234 | PHF3 | 2.32 | 0.0408 |
| LIPK | -1.62 | 0.01 | ESF1 | 2.01 | 0.0248 | ARID4A | 1.8 | 0.0412 |
| PIBF1 | 1.59 | 0.01 | UACA | 1.96 | 0.0253 | LPAR4 | 1.54 | 0.0424 |
| OR4F14P | 1.94 | 0.01 | JMJD1C | 1.99 | 0.0259 | GOLGA4 | 1.77 | 0.0424 |
| RB1CC1 | 2.46 | 0.0101 | BDP1 | 1.68 | 0.026 | NBEAL1 | 1.57 | 0.0429 |
| PWAR6 | 1.65 | 0.0113 | BOD1L1 | 2.05 | 0.0262 | ANKRD20A12P | 2.02 | 0.0433 |
| TAS2R31 | 2.23 | 0.0116 | BDP1 | 1.71 | 0.0263 | ANKRD20A12P | 1.84 | 0.0437 |
| TAS2R50 | 1.72 | 0.0116 | BAZ2B | 1.59 | 0.0266 | APC | 1.88 | 0.0445 |
| ZNF638 | 1.51 | 0.0122 | EEA1 | 2.39 | 0.027 | SAMD9L | 1.86 | 0.0454 |
| PPIG | 1.9 | 0.0123 | MNS1 | 1.73 | 0.0275 | TRIP11; ATXN3 | 2.21 | 0.0457 |
| HMGN5 | 1.56 | 0.0132 | CCDC14 | 1.51 | 0.0288 | MIS18BP1 | 2.46 | 0.0462 |
| REV3L | 1.83 | 0.0139 | N4BP2 | 1.83 | 0.0288 | PHIP | 1.82 | 0.0463 |
| ROCK2 | 1.8 | 0.0152 | ZBTB41 | 1.89 | 0.029 | VPS13A | 1.75 | 0.0464 |
| | | | | | | NIPBL | 2.16 | 0.0483 |

**Table S4.** Differential gene expression profile of U87 cells treated with 3$\alpha$-THP vs P4.

| 3$\alpha$-THP vs P4 | | | | | | | | |
|---|---|---|---|---|---|---|---|---|
| Gene Symbol | Fc | P-val | Gene Symbol | Fc | P-val | Gene Symbol | Fc | P-val |
| OR5M6P | 2.05 | 1.22E-05 | PYROXD1 | 1.58 | 0.0207 | KIF20B | 2.26 | 0.0377 |
| FMO8P | 2.38 | 0.0033 | KIAA1551 | 1.55 | 0.021 | PIBF1 | 1.6 | 0.0378 |
| GJB7 | -1.5 | 0.0124 | USP53 | 1.86 | 0.0221 | CHML | 1.75 | 0.0385 |

| | | | | | | | | |
|---|---|---|---|---|---|---|---|---|
| NSRP1 | 1.66 | 0.0138 | TPR | 1.95 | 0.0294 | N4BP2 | 1.71 | 0.0391 |
| ESF1 | 2.13 | 0.0141 | BDP1 | 1.69 | 0.0298 | KTN1 | 1.85 | 0.0393 |
| SNORA1; TAF1D; SNORA8 | 1.68 | 0.0154 | BOD1L1 | 1.73 | 0.0301 | ZNF480 | 1.61 | 0.0401 |
| ZEB2 | 1.7 | 0.0161 | REV3L | 1.51 | 0.0322 | OPN1LW | 1.51 | 0.0417 |
| CCDC18 | 1.82 | 0.0161 | RIF1 | 2.05 | 0.0326 | RB1CC1 | 1.68 | 0.0425 |
| RNU6-14P | 1.7 | 0.0166 | BDP1 | 1.68 | 0.0327 | FAM99A | -1.61 | 0.0427 |
| RAD50 | 1.7 | 0.0166 | ZBTB41 | 1.82 | 0.0334 | GSTT1 | -1.51 | 0.0452 |
| ROCK1 | 1.54 | 0.0169 | CCDC88A | 2.33 | 0.0355 | TAS2R14 | 1.52 | 0.0458 |
| LRRCC1 | 1.86 | 0.0203 | EIF5B | 1.88 | 0.0367 | VPS13A | 1.72 | 0.0489 |

**Table S5.** Differential gene expression profile of U87 cells treated with 3$\alpha$-THP vs F.

| **3$\alpha$-THP vs F** | | | | | |
|---|---|---|---|---|---|
| **Gene Symbol** | **Fc** | **P-val** | **Gene Symbol** | **Fc** | **P-val** |
| OR5M6P | 1.83 | 4.49E-05 | TRIM51CP | -1.68 | 0.0086 |
| KRTAP5-5 | 1.63 | 0.0006 | PRDM9 | -1.63 | 0.0392 |
| | | | SUMO1P3 | -1.65 | 0.0396 |

**Table S6.** Differential gene expression profile of U87 cells treated with P4 vs F.

| **P4 vs F** | | | | | | | | |
|---|---|---|---|---|---|---|---|---|
| **Gene Symbol** | **Fc** | **P-val** | **Gene Symbol** | **Fc** | **P-val** | **Gene Symbol** | **Fc** | **P-val** |
| ARHGAP23 | 1.6 | 0.0047 | RBMY1B; RBMY1D; RBMY1E | -1.59 | 0.0255 | RPSAP58 | 1.76 | 0.0381 |
| TRIM51CP | -1.58 | 0.0148 | RAD50 | -1.59 | 0.035 | HIST1H2BL | 1.51 | 0.0385 |
| LOC10106025 6 | 1.6 | 0.0166 | ROCK1 | -1.63 | 0.0354 | ANKRD20A8P; ANKRD20A12P | -1.54 | 0.0419 |
| | | | | | | PLPPR1 | 1.6 | 0.0495 |

**Table S7.** Genes chosen for validation.

| Gene full name and locus | Gene product characteristics and function |
|---|---|
| ESF1: ESF1 nucleolar pre-rRNA processing protein homolog<br><br>20p12.1 | ESF1 (also called ABTAP) codes for an evolutionarily conserved nucleolar protein that is part of a system that controls RNA polymerase II-directed transcription. It is involved in the suppression of gene expression by forming a complex with the transcription factor ABT1 [1]. Depletion of the ortholog of this gene in *S. cerevisiae* (Ydr365cp) results in a significant loss of the 18S rRNA, arguing that this protein has discrete domains that mediate direct catalytic functions in the pre-rRNA processing [2]. Furthermore, the functions of nucleolar proteins are tightly linked to cell growth and proliferation [3]. |
| TPR: Translocated promoter region, nuclear basket protein<br><br>1q31.1 | A protein that forms intracellular filaments anchored to the inner surface of the nuclear pore complex, it forms dimers through its N-terminal domain. TPR is required for the export of mRNA and proteins from the nucleus to the cytoplasm [4]. In the HeLa cell line, TPR silencing promotes cell cycle arrest in $G_0/G_1$ phase, the appearance of a senescent phenotype, and the accumulation of P53 in the nucleus [5]. |
| RIF1: Replication timing regulatory factor 1 | This gene encoding the RIF1 protein was first characterized in yeasts and later was found to be highly conserved among species [6]. RIF1 positively regulates DNA replication [7] and participates in double-strand DNA repair. During anaphase, RIF1 is recruited mainly to the centromeric region, where DNA |

| | |
|---|---|
| 2q23.3 | strand remains concatenated before topoisomerase II separates the chains. The silencing of RIF1 is associated with micronuclei formation [8]. Also, it is located in aberrant telomeres to promote the repair of the damage [9]. |
| RAD50: RAD50 double-strand break repair protein<br><br>5q31.1 | The protein encoded by this gene is one of the three components of the MRN complex (Mre11/Rad50/NBS1). The ATPase-nuclease Mer11 and Rad50 are evolutionarily conserved in all organisms [10]. The MRN complex plays a central role in the repair of DNA double-stranded breaks (DSBs), either by homologous recombination (HR) or non-homologous end joining (NHEJ) binding. It also participates in activating the cell cycle ATM checkpoints, the maintenance of telomeres, and meiotic recombination [11]. Mutations in members of the MRN complex are associated with severe phenotypes of cancer susceptibility. In RAD50s/s mice, germline and hematopoietic cell deficiencies are the results of stem cell decay, promoting defects in animal growth, the presence of lymphomas and predisposition to cancer [12,13]. Besides, it has also been found that the expression of RAD50 is increased in melanoma [14]. |
| ROCK1: Rho-associated coiled-coil containing protein kinase 1<br><br>18q11.1 | This gene encodes for a serine/threonine kinase that keeps 65% of identity with ROCK2 kinase and 92% with the kinase domain of this protein [15]. ROCK1 is an effector of the Rho GTPase and is involved in the phosphorylation of many substrates such as the cytoskeleton protein myosin [16]. Overexpression of this gene is correlated with an increase in cell proliferation, invasion, migration, and metastasis of human lung and breast cancer cell lines as well as GBMs [17–19]. |
| ROCK2: Rho-associated coiled-coil containing protein kinase 22p25.1 | The protein encoded by this gene is a serine/threonine kinase of the same family as ROCK1. It regulates cytokinesis, the formation of actin fibers, and the activation of c-fos response factor. It is associated with actin microfilaments as well as with the stress fibers of growing cells [19,20]. Contrary to ROCK1, ROCK2 is mainly expressed in the CNS [21]. |
| REV3L: REV3-like, DNA directed polymerase zeta catalytic subunit 6q21 | REV3L is the orthologous gene of the *S. cerevisiae* REV3 gene encoding the catalytic subunit of the DNA polymerase $\zeta$. It is highly conserved in mammals and is indispensable during development, its deletion in mice causes embryonic death [22]. DNA polymerase $\zeta$ is a heterotetramer constituted by a REV3 heterodimer, REV7 and two accessory proteins that are shared with the Pol $\delta$ [23]. Implicated in double-strand DNA break repair, lacks 3′-5′exonuclease activity and maintains the genome integrity at the expense of the introduction of mutations in the genome. Hence, it is associated with the tumorigenic potential of the cells and/or the progression of cancer [24]. Besides, the conditional knockout mice for REV3L show spontaneous tumors [25]. |
| PCM1: Pericentriolar material 1 protein<br><br>8p22 | The protein encoded by this gene is a major component of the pericentriolar satellites that determine the centrosomes. It remains attached to the centrosome during $G_1$, S, and at the beginning of the $G_2$ phase [26]. PCM1 is essential for the localization of centrosomal proteins and the anchorage of the centrosome microtubules since it recruits the Pkl1 kinase to the pericentriolar material during primary cilia disassembly [27]. Chromosomal aberrations of this gene have been associated with leukemia and pre-menopausal breast cancer [28]. |
| DYNC2H1: Dynein cytoplasmic 2 heavy chain 1<br><br>11q22.3 | This gene encodes for the heavy chain 1 of the dynein 2 and is found highly conserved among vertebrates, and is widely expressed in ciliated cells. The protein forms homodimers and associates with the light chain intermediated DYNC2LI1 [29]. Has ATPase activity and is involved in the cilia and flagella retrograde transport [30]. Also, it may participate in the protein transport in the Golgi complex, but this has not been fully elucidated [31]. |
| CCDC91: Coiled-coil domain | This protein participates in the protein transport in the Golgi complex. It interacts with monomeric GGA proteins (Golgi-localized, $\gamma$-Ear-containing, |

| | |
|---|---|
| containing 91, P56 protein 12q11.22 | ADP-ribosylation factor-binding proteins) [32] regulating the trans-Golgi network and endosomal transport [33,34]. It is widely expressed in neuroblastoma, neuroepithelial, melanoma, and fibroblast cell lines. |

**References**


1. Oda, T.; Fukuda, A.; Hagiwara, H.; Masuho, Y.; Muramatsu, M. A.; Hisatake, K.; Yamashita, T. ABT1-associated protein (ABTAP), a novel nuclear protein conserved from yeast to mammals, represses transcriptional activation by ABT1. *J. Cell. Biochem.* **2004**, *93*, 788–806, doi:10.1002/jcb.20114.

2. Peng, W. T.; Krogan, N. J.; Richards, D. P.; Greenblatt, J. F.; Hughes, T. R. ESF1 is required for 18S rRNA synthesis in Sccharomyces cerevisiae. *Nucleic Acids Res.* **2004**, *32*, 1993–1999, doi:10.1093/nar/gkh518.

3. Andersen, J. S.; Lam, Y. W.; Leung, A. K. L.; Ong, S.-E.; Lyon, C. E.; Lamond, A. I.; Mann, M. Nucleolar proteome dynamics. *Nature* **2005**, *433*, 77–83, doi:10.1038/nature03207.

4. Ben-Efraim, I.; Frosst, P. D.; Gerace, L. Karyopherin binding interactions and nuclear import mechanism of nuclear pore complex protein Tpr. *BMC Cell Biol.* **2009**, *10*, 74, doi:10.1186/1471-2121-10-74.

5. David-Watine, B. Silencing Nuclear Pore Protein Tpr Elicits a Senescent-Like Phenotype in Cancer Cells. *PLoS One* **2011**, *6*, e22423, doi:10.1371/journal.pone.0022423.

6. Smogorzewska, A.; de Lange, T. Regulation of telomerase by telomeric proteins. *Annu. Rev. Biochem.* **2004**, *73*, 177–208, doi:10.1146/annurev.biochem.73.071403.160049.

7. Hiraga, S.; Ly, T.; Garzón, J.; Hořejší, Z.; Ohkubo, Y.; Endo, A.; Obuse, C.; Boulton, S. J.; Lamond, A. I.; Donaldson, A. D. Human RIF1 and Protein Phosphatase 1 stimulate DNA replication origin licensing but suppress origin activation. *EMBO Rep.* **2016**, *18*, 403–419, doi:10.15252/embr.201641983.

8. Hengeveld, R. C. C.; de Boer, H. R.; Schoonen, P. M.; de Vries, E. G. E.; Lens, S. M. A.; van Vugt, M. A. T. M. Rif1 Is Required for Resolution of Ultrafine DNA Bridges in Anaphase to Ensure Genomic Stability. *Dev. Cell* **2015**, *34*, 466–474, doi:10.1016/j.devcel.2015.06.014.

9. Xu, L.; Blackburn, E. H. Human Rif1 protein binds aberrant telomeres and aligns along anaphase midzone microtubules. *J. Cell Biol.* **2004**, *167*, 819–830, doi:10.1083/jcb.200408181.

10. Hopfner, K. P. ATP puts the brake on DNA double-strand break repair: A new study shows that ATP switches the Mre11-Rad50-Nbs1 repair factor between signaling and processing of DNA ends Prospects & Overviews K.-P. Hopfner. *BioEssays* **2014**, *36*, 1170–1178, doi:10.1002/bies.201400102.

11. van den Bosch, M.; Bree, R. T.; Lowndes, N. F. The MRN complex: coordinating and mediating the response to broken chromosomes. *EMBO Rep.* **2003**, *4*, 844–9, doi:10.1038/sj.embor.embor925.

12. Bender, C. F.; Sikes, M. L.; Sullivan, R.; Huye, L. E.; Beau, M. M. Le; Roth, D. B.; Mirzoeva, O. K.; Oltz, E. M.; Petrini, J. H. J. Cancer predisposition and hematopoietic failure in Rad50 S / S mice. *Genes Dev.* **2002**, *16*, 2237–2251, doi:10.1101/gad.1007902.like.

13. de Jager, M.; Kanaar, R. Genome instability and Rad50(S): subtle yet severe. *Genes Dev.* **2002**, *16*, 2173–2178, doi:10.1101/gad.1025402.

14. Avaritt, N. L.; Owings, R.; Reynolds, M.; Larson, S. K.; Byrum, S.; Hiatt, K. M.; Smoller, B. R.; Tackett, A. J.; Cheung, W. L. Misregulation of Rad50 expression in melanoma cells. *J. Cutan. Pathol.* **2012**, *39*, 680–684, doi:10.1111/j.1600-0560.2012.01915.x.

15. Chin, V. T.; Nagrial, A. M.; Chou, A.; Biankin, A. V; Gill, A. J.; Timpson, P.; Pajic, M. Rho-associated kinase signalling

and the cancer microenvironment: novel biological implications and therapeutic opportunities. *Expert Rev. Mol. Med.* **2015**, *17*, e17, doi:10.1017/erm.2015.17.

16. Julian, L.; Olson, M. F. Rho-associated coiled-coil containing kinases (ROCK): structure, regulation, and functions. *Small GTPases* **2014**, *5*, e29846, doi:10.4161/sgtp.29846.

17. Zohrabian, V. M.; Forzani, B.; Chau, Z.; Murali, R.; Jhanwar-Uniyal, M. Rho/ROCK and MAPK signaling pathways are involved in glioblastoma cell migration and proliferation. *Anticancer Res.* **2009**, *29*, 119–23.

18. Li, B.; Zhao, W.-D.; Tan, Z.-M.; Fang, W.-G.; Zhu, L.; Chen, Y.-H. Involvement of Rho/ROCK signalling in small cell lung cancer migration through human brain microvascular endothelial cells. *FEBS Lett.* **2006**, *580*, 4252–4260, doi:10.1016/j.febslet.2006.06.056.

19. Hsu, C.-Y.; Chang, Z.-F.; Lee, H.-H. Immunohistochemical evaluation of ROCK activation in invasive breast cancer. *BMC Cancer* **2015**, *15*, 943, doi:10.1186/s12885-015-1948-8.

20. Katoh, K.; Kano, Y.; Noda, Y. Rho-associated kinase-dependent contraction of stress fibres and the organization of focal adhesions. *J. R. Soc. Interface* **2011**, *8*, 305–311, doi:10.1098/rsif.2010.0419.

21. Yin, H.; Hou, X.; Tao, T.; Lv, X.; Zhang, L.; Duan, W. Neurite outgrowth resistance to rho kinase inhibitors in PC12 Adh cell. *Cell Biol. Int.* **2015**, *39*, 563–576, doi:10.1002/cbin.10423.

22. Makarova, A. V.; Burgers, P. M. Eukaryotic DNA polymerase ζ. *DNA Repair (Amst).* **2015**, *29*, 47–55, doi:10.1016/j.dnarep.2015.02.012.

23. Baranovskiy, A. G.; Lada, A. G.; Siebler, H. M.; Zhang, Y.; Pavlov, Y. I.; Tahirov, T. H. DNA polymerase δ and ζ switch by sharing accessory subunits of DNA polymerase δ. *J. Biol. Chem.* **2012**, *287*, 17281–17287, doi:10.1074/jbc.M112.351122.

24. Zhu, X.; Zou, S.; Zhou, J.; Zhu, H.; Zhang, S.; Shang, Z.; Ding, W.-Q.; Wu, J.; Chen, Y. REV3L, the catalytic subunit of DNA polymerase ζ, is involved in the progression and chemoresistance of esophageal squamous cell carcinoma. *Oncol. Rep.* **2016**, *35*, 1664–1670, doi:10.3892/or.2016.4549.

25. Wittschieben, J. P.; Patil, V.; Glushets, V.; Robinson, L. J.; Kusewitt, D. F.; Wood, R. D. Loss of DNA polymerase zeta enhances spontaneous tumorigenesis. *Cancer Res.* **2010**, *70*, 2770–2778, doi:10.1158/0008-5472.CAN-09-4267.

26. Balczon, R.; Bao, L.; Zimmer, W. E. PCM-1, a 228-kD centrosome autoantigen with a distinct cell cycle distribution. *J. Cell Biol.* **1994**, *124*, 783–793, doi:10.1083/jcb.124.5.783.

27. Wang, G.; Chen, Q.; Zhang, X.; Zhang, B.; Zhuo, X.; Liu, J.; Jiang, Q.; Zhang, C. PCM1 recruits Plk1 to the pericentriolar matrix to promote primary cilia disassembly before mitotic entry. *J. Cell Sci.* **2013**, *126*, 1355–65, doi:10.1242/jcs.114918.

28. Armes, J. E.; Hammet, F.; de Silva, M.; Ciciulla, J.; Ramus, S. J.; Soo, W.-K.; Mahoney, A.; Yarovaya, N.; Henderson, M. a; Gish, K.; Hutchins, A.-M.; Price, G. R.; Venter, D. J. Candidate tumor-suppressor genes on chromosome arm 8p in early-onset and high-grade breast cancers. *Oncogene* **2004**, *23*, 5697–702, doi:10.1038/sj.onc.1207740.

29. Mikami, A.; Tynan, S. H.; Hama, T.; Luby-Phelps, K.; Saito, T.; Crandall, J. E.; Besharse, J. C.; Vallee, R. B. Molecular structure of cytoplasmic dynein 2 and its distribution in neuronal and ciliated cells. *J Cell Sci* **2002**, *115*, 4801–4808, doi:10.1242/jcs.00168.

30. Pfister, K. K.; Shah, P. R.; Hummerich, H.; Russ, A.; Cotton, J.; Annuar, A. A.; King, S. M.; Fisher, E. M. C. Genetic analysis of the cytoplasmic dynein subunit families. *PLoS Genet.* **2006**, *2*, 11–26, doi:10.1371/journal.pgen.0020001.

31. Vaisberg, E. A.; Grissom, P. M.; Mclntosh, J. R. Mammalian cells express three distinct dynein heavy chiains that are

colalized to different cytoplasmic organelles. **1996**, *133*, 831–842.

32. Mardones, G. A.; Burgos, P. V.; Brooks, D. A.; Parkinson-Lawrence, Emma Mattera, R.; Bonifacino, J. S. The Trans-Golgi Network Accessory Protein p56 Promotes Long-Range Movement of GGA/Clathrin-containing Transport Carriers and Lysosomal Enzyme Sorting. *Mol. Biol. Cell* **2007**, *18*, 3486–3501, doi:10.1091/mbc.E07.

33. Bonifacino, J. S. The GGA proteins: adaptors on the move. *Nat Rev Mol Cell Biol* **2004**, *5*, 23–32, doi:10.1038/nrm1279.

34. Ghosh, P.; Kornfeld, S. The GGA proteins: key players in protein sorting at the trans-Golgi network. *Eur. J. Cell Biol.* **2004**, *83*, 257–262, doi:10.1078/0171-9335-00374.
